# Repeated Intrathecal Triamcinolone Acetonide Administration in Progressive Multiple Sclerosis: A Review

**DOI:** 10.1155/2011/219049

**Published:** 2011-06-26

**Authors:** Mazen Abu-Mugheisib, Reiner Benecke, Uwe K. Zettl

**Affiliations:** Department of Neurology, University of Rostock, Gehlsheimer Straße 20, 18147 Rostock, Germany

## Abstract

At the present time, anti-inflammatory, immunomodulatory, or immunosuppressive treatments of multiple sclerosis (MS) are mainly effective in the early phases of the disease but are of less advantage in progressive phases. Current therapeutic strategies of both primary and secondary progressive MS are rare. One alternative may be intrathecal application of triamcinolone acetonide (TCA). Number of papers deal with advantages and disadvantages of intrathecal administration in MS. Former trials lacked detailed selection of MS patients, with small sample sizes, low steroid dosages, and only a small number of intrathecal administration of short acting steroids. The present paper summarizes recent trials performed following a different treatment regime. They were conducted in patients with progressive MS suffering mainly from spinal symptoms and documented a significant improvement of EDSS and walking distance (WD). Intrathecal TCA administration is a proposal to take into account as one therapy option in patients with a progressive clinical course and predominantly spinal symptoms.

## 1. Introduction

Multiple sclerosis (MS) as one of the most frequent diseases of the central nervous system (CNS) in young adults often entails persistent physical and mental disability. The prevalence is assumed to be 400,000 people in the United States and approximately 2,1 million people worldwide [1]. MS is an immune-mediated demyelinating inflammatory disease. Its natural history in most patients is marked by a chronic progressive decline [2]. Mostly, MS begins with a relapsing course (relapse remitting MS, RRMS). After years, it leads to a progressive course (secondary progressive MS, SPMS) [3, 4]. Another form of MS, progressive-relapsing MS (PRMS), is defined as a progressive disease from onset with acute relapses and with periods of progression between relapses. Nearly 10% of MS patients develop primary progressive MS (PPMS), which is defined as a progressive type from onset with temporary impairment. Regarding to the pathogenesis of MS, two different approximations are common. MS is mainly characterized by multitopic inflammation and demyelination. As the disease proceeds, the role of axonal loss and gliosis increases [5].

Hence, MS pathophysiology is much more complex than assumed up to now. Consequently, one therapy with a single immune mechanism cannot fit such a complex pathogenic disease. At the present time, the anti-inflammatory, immunomodulatory, or immunosuppressive treatments are mainly effective in the early phase of the disease but are of less advantage in the progressive phase [6]. Therefore, an axon-protective therapy will be essential to reduce disease progression [7]. Current treating strategies of progressive MS are rare. Mitoxantrone is an FDA-approved therapy option for progressive phase in MS. Meanwhile, the application of glucocorticosteroids in the treatment of relapses has been accepted. There is general agreement that intravenous methylprednisolone (IVMP) administration (1000 mg daily for 3–5 days) is first-line therapy in the recovery from relapses [8–10]. Treatment with IVMP minimizes tissue damage and assists lesion recovery in patients with RRMS [11]. IVMP recovers the blood-brain barrier (BBB) by downregulating adhesion molecule expression. Furthermore, it induces different immunological changes as inhibition of proinflammatory cytokines, lymphocyte apoptosis, and remyelination [12]. So, as of a result, their immunosuppressive and anti-inflammatory power glucocorticoids are established in the standard treatment for acute relapses. Although IVMP could reduce the duration of a relapse, no effect on the exacerbation rate or on the development of long-term disability was determined [13]. The benefit of corticosteroids in the treatment of acute relapses has been examined in clinical trials. Another double-blind, placebo-controlled, randomized trial of high-dose methylprednisolone (1 g IV daily for 5 days) was arranged in 35 patients with PPMS [14]. A statistically significant amelioration of the expanded disability status scale (EDSS) score was observed. This improvement persisted for at least 3 months. One phase II randomized controlled trial (RCT) in RRMS compared the benefit of repeated pulsed IVMP with IVMP at the same dosage but administered only for relapses. It could be demonstrated that pulsed IVMP decreased the development of T1 black holes, brain atrophy progression, and associated development of permanent disability [10]. On the other hand, pulsed application of intravenous corticosteroids is related to transient and dose-dependent side effects, such as temporary mood disorders, gastric ulcer, headache, and myalgia [15]. Chronic administration may even result in more serious side effects, such as hypertension, hyperglycemia, decline of cardiac conditions, osteoporosis and an increased incidence of fractures, hepatic steatosis, infection, cataract, and transient memory impairment [16]. Consequentially, one interesting alternative may be the intrathecal administration of triamcinolone acetonide (TCA), which has been adopted for the treatment of many other diseases. This paper reviews data on the efficacy of intrathecal steroid application in the treatment of MS. Trials were classified according to the system established by the American Academy of Neurology (AAN) [17].

## 2. Historical Experiences with Intrathecal Steroids in Multiple Sclerosis

A number of historical papers deal with advantages and disadvantages of intrathecal administration in MS. Since 1953, several mainly uncontrolled trials have been published. Different dosages and diverse conventional steroid compounds, that is, methylprednisolone acetate (MPA) or TCA, are mentioned. Despite the controversial discussion especially in progressive MS patients with predominantly spinal symptomatology according to some trials, positive effects could be noticed [18–20] (Table 1).

In 1953, Kamen and Erdman [23] referred treating a patient with RRMS with intrathecal hydrocortisone (HC) and intramuscular adrenocorticotropic hormone (ACTH). The patient recovered during a 6-week follow up. In a couple of open-label, uncontrolled trials between 1961 and 1963, Boines [24, 25] reported 75–80% recovery, particularly of spasticity with intrathecal MPA in 42 patients during a follow up of 12–52 weeks. Goldstein et al. [30] reported that intrathecal MPA decreased CSF *γ*-globulin in MS but without correlation to improvement of spasticity. In 1964, Van Buskirk et al. [26] performed an open-label, uncontrolled prospective study of intrathecal MPA in 20 patients. The treatment appears to decrease spasticity in 14 patients and consequently results in improved walking distance and bladder function. In 1970, again Goldstein et al. [27] referred in an open-label, uncontrolled trial to 38 patients treated with 4 to 8 intrathecal MPA infusions and followed up for 2 to 8 years. Neurological examinations revealed an initial improvement in 30 patients that remained stable in only 6 patients. In 1973, again Nelson et al. [28] reported in an open-label, uncontrolled prospective study on 23 patients with MS. They received intrathecal MPA infusions for acute exacerbations. A mild amelioration of EDSS was detected in 4 patients (17%). All the above-mentioned studies have to be rated as class IV evidence, only.

For the first time, Rohrbach et al. [29] performed a double-blind, randomized, controlled trial (short report). 42 distinctly chronic progressive MS patients with predominantly spinal symptoms were enrolled. One cohort was treated either with 3 or 4 intrathecal TCA injections of 80 mg. The other cohort received oral triamcinolone starting with 48 mg/d in descending dosage. In the intrathecal cohort, a better and consistent improvement in the spinal score could be observed than the other treatment arm. This study corresponds to class II evidence. 

In 1992, Heun et al. [18] conducted an open-label, randomized, prospective, unblinded study on 50 MS patients with different MS forms (RRMS, PPMS, and SPMS). One group received 3 intrathecal TCA injections of 40 mg on days 1, 8, and 15; the other cohort was treated with methylprednisolone 100 mg i.v. from day 1 to 5, then in descending dose. A slender but significant improvement in disability was noticeable in both cohorts. No significant difference in the examined frequency of improved neurological symptoms or in EDSS between the two cohorts was found. The study has to be classified as class III evidence. 

In conclusion, the majority of the mentioned historical trials of intrathecal steroid for MS performed in the past were uncontrolled and have to be rated as class IV evidence. Despite their lacks, the trials of Rohrbach et al. [29] and Heun et al. [18] are notable (class II/III evidence). Especially trials that conform to generally defined criteria of evidence-based medicine are missing. According to intrathecal TCA applications, there is a controversial discussion [19, 31]. Repeated lumbar punctures under double-blind design including the agreement of patients and the ethical committee are nowadays not feasible.

### 2.1. Risks

Intrathecal MPA therapy for MS caused transient urinary incontinence in two of 20 patients [26]. In two other reports on 61 patients, constrictive arachnoiditis in thoracic or lumbar area, aseptic meningitis, subarachnoid haemorrhage, and neurogenic bladder were described [27, 28]. Other mentioned complications were brain damage, spinal cord lesions, and dense widespread pachymeningitis [32–34]. In spite of these reports, intrathecal steroid therapy is still advised [21, 35]. 

## 3. Recent Trials with Intrathecal TCA Administration in Progressive Multiple Sclerosis

The revival of intrathecal steroid treatment started with the positive results of a trial on intractable postherpetic neuralgia, in which 89 subjects received up to 4 intrathecal methylprednisolone administrations within 4 weeks without any serious side effects [36]. In a rapid succession, a few further open-label uncontrolled trials were performed following a different treatment regime [37–39] (Table 2).

Hoffmann et al. [37] performed an open-label, uncontrolled, prospective trial on the short-term and long-term efficacy and tolerability of repeated intrathecal TCA application. 36 patients with progressive MS (22 SPSS, 14 PPMS, EDSS < 7.5) were included. Patients did not receive steroids and were on a stable immunomodulatory drug treatment for at least 4 weeks before the start of the study. They had to show symptom progression of at least one point on the EDSS scale, in the last 2 years before study entry, but had to be stable for at least 4 weeks before inclusion. An atraumatic (Sprotte^®^) needle was used in order to minimize the risk of postlumbar puncture syndrome [41]. 6 injections with 40 mg TCA were administered within 3 weeks. EDSS scores significantly decreased (*P* = .00065), and the walking distance (WD) significantly increased (*P* = .003). None of the measured parameters deteriorated in any patient. Patients with an improvement in their EDSS or WD were provided to receive further treatment with one TCA application at an individual rate every 6 to 12 weeks. The follow-up treatment period amounted to 13.1 ± 6.22, 3–23 (mean ± S.D., range) months with 6.35 ± 3.91, 2–15 TCA administrations. The post hoc analysis demonstrated that a significant decline of EDSS and the improvement of WD occurred after first initial 6 TCA applications and then remained stable. Neither a significant impact of covariates in statistical analysis nor relevant side effects were found. This study accomplished a total of 340 lumbar punctures. A temporary increase of CSF protein above 500 mg/L and transitory increase of CSF cells (maximum cell count was 38/*μ*L) was noticed. Nevertheless, no new clinical symptoms were caused in any subject. 5 patients developed a slight post-lumbar puncture syndrome, but they did not abandon further TCA applications. This study illustrated efficacy and safety of repeated intrathecal TCA administration in progressive MS patients with spinal symptoms. The application frequency (6 TCA injections within 3 weeks and follow-up injection every 6 to 12 weeks) was markedly higher in contrast to other previous trials. This analysis demonstrated that particularly PPMS and SPMS patients benefit from described therapy design. Although long-term data did not prove any further improvement of neurological symptoms, the amelioration reached remained robust over the following treatment period with one TCA application every 6 to 12 weeks. Nevertheless, this uncontrolled study has to be graduated as class IV evidence. 

Hellwig et al. [38] performed another open-label, uncontrolled, prospective study on 161 MS patients (35 PPMS, 122 SPMS, 4 RRMS) with pronounced spinal symptoms on the impact of the administration of 40 mg of the sustained released steroid TCA. Subjects did not suffer from an acute onset of exacerbation or recent pronounced increased progression of MS symptoms. An established immune system modulating therapy was not altered. EDSS, Barthel index, WD, and somatosensory evoked potentials (SSEPs) were analysed before start and at the end of the TCA treatment [42]. The patients achieved a supplemental standardized rehabilitation therapy. Atraumatic Sprotte^®^ needles were used to avoid post-lumbar puncture syndrome [41, 43]. Each patient received 6 applications of 40 mg TCA within 3 weeks. EDSS and Barthel indices were enhanced, WD increased, and latencies of SSEP of the median and tibial nerves were reduced in all patients at serial evaluation (*P* < .0001 for all variables). Neither slight nor severe side effects were registered. 5 patients abandoned the study due to lumbar puncture headache.

In this uncontrolled trial, an improvement of spinal symptoms, WD, and SSEP latencies in progressive MS patients were documented, and the results from a previous trial were confirmed [37]. The electrophysiological results may mirror a certain potential of intrathecal TCA administration for demyelinating actions. Again this uncontrolled study has to be rated as class IV evidence.

### 3.1. Absent Hints as to Cell Injury by Repeated TCA Applications

Steroids were suspected to induce a neuronal cell injury due to brain atrophy [10, 22, 44]. Another open-label, uncontrolled, prospectice trial on short-term efficacy of repeated intrathecal TCA applications in progressive MS dealt with this aspect [39]. 27 subjects with progressive MS were included. They received similar therapy as described in previous trials [37, 38]. In addition to the mentioned clinical parameters, CSF was examined for the unspecific markers of cell injury neuron-specific enolase (NSE), Tau-protein, S 100B, and *β*-amyloid [45–49]. 6 TCA injections, performed every third day, reduced EDSS (initial: 5.4 ± 1.3, 3–7.5 (mean ± SD, range); end: 4.9 ± 1.1; 2.5–6.5; *P* < .001) and significantly increased WD primarily after the fourth TCA injection. These results indicated that the role of TCA administrations is undercharged in those trials without any persuasive clinical output [18, 20]. The assessed CSF marker did not significantly change within the interval of TCA treatment. This supported the statement that the sustained released steroid TCA is not toxic and causes no relevant cell injury or deterioration of neuronal cells [10, 20, 44, 50–53]. Furthermore, no serious clinical side effects appeared. This uncontrolled study has to be classified as class IV evidence. 

#### 3.1.1. Comparison of Repeated Intrathecal TCA Administration with Mitoxantrone Therapy in Patients with Progressive MS

Previous studies showed that repeated intrathecal TCA administrations generated a clear prolonged benefit in patients with progressive MS suffering from mainly spinal symptoms [37]. Mitoxantrone (MIX) application is performed similarly in progressive MS patients with a continuous, rapid worsening of symptoms [54]. In contrast to TCA administration, MIX application is a worldwide accredited therapy to diminish or abandon progression. There exists important restriction due to its cardiac toxicity. Hence, a cumulative maximal life-time dose should be respected [54–56]. 

Based on this consideration, Hellwig et al. [40] performed an open-label study over a 52-week-long interval and compared TCA and MIX therapy in two matched cohorts of subjects with progressive MS. Only patients with progressive MS with an EDSS ≤ 7.5 were recruited. In the MIX arm, 30 patients were included and observed over 1 year. The initial MIX dose was 12 mg/m^2^. The second infusion was followed 6 weeks later and then quarterly. The MIX dose was minimized to 10 mg/m^2^ and 8 mg/m^2^ dependent on patients' stable condition. 34 patients were recruited in the TCA arm and treated as previously described [37]. EDSS significantly decreased and WD significantly increased (*P* < .001) after the initial 6 TCA administrations and then remained relatively constant. Neither EDSS nor WD deteriorated in any of the TCA patients. On the other hand, MIX therapy did not significantly influence EDSS (*P* = .056) or WD (*P* = .12), even though no additional decline of EDDS or WD was measured. Two patients in the MIX arm suffered from moderate nausea. An isolated and temporary increase in CSF protein (>500 mg/L) and a temporary rise of CSF cells without development of neurological symptoms in all subject was observed. 8 patients in the TCA arm suffered from post-lumbar puncture syndrome without termination of further TCA treatment. Again, the efficacy and safety of repeated intrathecal TCA administrations in progressive MS patients with predominantly spinal symptoms was approved [37, 38]. It has to be pointed out that a rate with 6 TCA applications within 3 weeks was definitively higher compared to previous trials [18]. Following this concept, especially PPMS and SPMS patients appear to improve initially and then remain stable during TCA treatment at least over one year. In contrast in this trial, MIX therapy did not improve EDSS or WD, but no significant impairment was recognized. Other trials approved a positive impact of MIX on MS symptoms especially in patients with progressive MS and superposed relapses [54, 57, 58]. The number of relapses in the year before MIX treatment started is regarded to be a predictive parameter in MIX efficacy in MS patients [59]. In this trial, mainly patients without superimposed relapses were included. Therefore, the lack of EDSS improvement could be attributed to this. In conclusion, TCA and MIX proved their efficacy in different ways. Maybe a combination of both should be investigated in progressive MS patients as it has been performed with IVMP and MIX [60]. Despite the mentioned limitations, this study has to be rated as class III evidence. 

## 4. Conclusion

Up to now, clinical trials on patients with progressive MS demonstrated no distinct proof of a potent symptomatic treatment intended to improve or at least stabilize disability, as soon as the progressive phase of the disease stage appears. Immunomodulatory treatment minimizes the rate of MS relapses noticeably but shows no evident positive effects in patients with progressive MS [17]. So the immunomodulatory treatment is a rather preventive one. Numerous papers dealt with the efficacy of intrathecal application of different dosages of various released steroid compounds, above all methylprednisolone acetate was used. This formerly used steroids were mainly short acting cortisone derivates. Further, these steroids were administrated intrathecally less frequently. So, these trials lacked of detailed selection and clinical characterization of MS patients, with small sample sizes, low steroid dosages, and only a few intrathecal administration of mostly short-acting cortisone derivates [19, 20, 36]. Beneficial but controversialy discussed effects were mentioned in progressive MS patients with predominantly spinal symptoms according to case reports, open-label trials, and one double-blind, controlled study (class of evidence II) with the sustained released steroid TCA [18–20]. As is known, the anti-inflammatory impact of a steroid application depends not only on the dosage but also on the duration of exposure [9, 10, 61]. Hence, the frequency of application and the utilization of a delayed released steroid derivative as TCA are recommendable. 

In a rapid succession, a few further open-label uncontrolled trials were performed following a different treatment regime [37–39]. 6 injections of 40 mg of the sustained released steroid derivate TCA were administered within 3 weeks. Patients with an improvement of EDSS or WD were provided to receive further treatment with one TCA application in an individual rate every 6 to 12 weeks. All forecited more recent open label trials were performed in patients with progressive MS with mainly spinal symptoms. They documented a significant improvement of EDSS and WD, respectively. With additional administrations, a stable effect was achieved. However, the mechanism which these improvements are based on is unacquainted. One item debated was the decrease of spasticity by the long-acting steroid. But a significant decrease of antispastic scores was not essential to achieve the mentioned results in recent trials. Another point of discussion could be that intrathecal administration of a sustained released steroid circumvents the BBB and has a positive impact on the still continuing chronic inflammation process. The one thing common to all the recent examples we gave was that they had all been focused on progressive MS patients without signs of an acute exacerbation. 

The before-described great number of serious side effects could not be reproduced in the recent trials. There were some raised concerns about a possible neuronal cell injury promoting effect induced by the administered steroid, with inducing brain atrophy [10, 22, 44]. The additional serial assessment of potential unspecific cell injury markers, that is, NSE or S-100, in CSF of progressive MS patients treated with repeated intrathecal TCA did not provide evidence of such a steroid associated risk [39]. Particularly, the long half-life of the applied sustained released steroids appears to be the key of the missing proof of a toxic effect. Further detailed trials with examination of selected CSF biomarkers in MS patients treated with intrathecal steroids are necessary to illuminate these interesting aspects. 

In general, further trials are needed to gain more results about the utility of this therapy. All of the mentioned historical and recent studies have to be classified just as class II–IV evidence. The ideal trial design would be a randomized, placebo-controlled, double-blind one. In this case, repeated performance of intrathecal placebo application under double-blind conditions with the consent of patients and the ethical committee seems not to be realistic. Furthermore, such a design including withholding treatment causes maybe ethical qualms [62]. Contrariwise, one could claim that due to the limited evidence for efficacy of intrathecal TCA treatment, the only existing open-label, not placebo-controlled study with repeated lumbar punctures is unethical without level A evidence. 

From this point of view, further trials on the potency and safety of intrathecal TCA applications are needed. A multicenter clinical study has to be established to evaluate these items and to compare systemic and intrathecal steroid treatment, initially in progressive MS patients with predominantly spinal symptoms and afterwards in patients with an acute relapse. In addition to the investigation of the long- and short-term benefits, potential risks related to the intrathecal application have to be examined in a blinded analysis. Furthermore, the potential efficacy of intrathecal TCA treatment combined with MIX in progressive MS has to be explored [60]. 

Anyhow, the intrathecal TCA administration has to be taken into account as one therapy option in handpicked MS patients with a slow progressive clinical course with predominantly spinal symptom features. The intrathecal TCA application should be offered by neurologists with a comprehensive experience in this special treatment. In fact, an individual risk-benefit analysis and the patient's approval are required.

## Figures and Tables

**Table 1:** Representative intrathecal steroid trials 1953–1992 [21, 22].

	Design	Patients included, MS type	Dosage and duration	Primary outcome	Results	Evidence
Kamen and Erdman, 1953 [23]	Case report	1; RR	Intrathecal HC and intramuscular ACTH; no specific data available	Recovery	Patient recovered	IV

Boines, 1961 and 1963 [24, 25]	Open-label, uncontrolled, retrospective, unblinded follow up of 12–52 weeks	42; no specific data available	40–100 mg intrathecal MPA every 2-3 weeks for a total of 6 injections, then “follow-up booster injections”	“recovery, particular of spasticity”; no specific outcome data available	“80% of patients improved/showed excellent or good results”	IV

Van Buskirk et al., 1964 [26]	Open-label, uncontrolled, prospective, unblinded	20; no specific data available	Weekly increasing doses intrathecal MPA (20–80 mg), then booster injection monthly (80–100 mg MPA); follow up 1 week-16–months	“clinical improvement”	“no effect on frequency of exacerbations, but improvement in spasticity in 14 patients”	IV

Goldstein et al., 1970 [27]	Open-label, uncontrolled, retrospective, unblinded	38; no specific data available	40–80 mg intrathecal MPA/4–8 times within 1-2 weeks; follow up 2–8 years	“improvement”	“79% improvement”	IV

Nelson et al., 1973 [28]	Open-label, uncontrolled, prospective, unblinded	23; RR, SP	40–120 mg intrathecal MPA/1–23 times within 2 months; follow up 1–84 months	EDSS CSF changes	EDSS: 4 patients (17%) improved; significant increase of CSF protein	IV

Rohrbach et al., 1988 [29]	double-blind, randomized, prospective	42, “mainly chronic progressive”	Intrathecal TCA: 80 mg/3-4 times within 14 days Oral TCA: 48 mg/d, tapering off	“spinal score”	Intrathecal TCA: “better improvement in the spinal score”	II

Heun et al., 1992 [18]	open-label, prospective, randomized, unblinded, follow up of 21 days	Intrathecal TCA: 25 Systemic MPA: 25	TCA: 40 mg on days 1, 8, and 15 MPA: 100 mg for 5 days, tapering off	EDSS AI SSEP	EDSS improved in both groups (*P* < .01); EDSS changes between both groups n.s.; AI n.s.	III

TCA: triamcinolone-acetonide acid; HC: hydrocortisone; ACTH: adrenocorticotropic hormone; MPA: methylprednisolone acetate; RR: relapsing-remitting MS; PP: primary chronic progressive MS; SP: secondary chronic progressive MS; MIX: mitoxantrone, EDSS: expanded disability status scale; WD: maximum walking distance; WT: maximum walking time; SSEP: somatosensory evoked potentials; AI: ambulation index; CSF: cerebrospinal fluid; n.s: non significant.

**Table 2:** Representative intrathecal steroid investigations in multiple sclerosis (since 2003).

	Design	Patients included, MS type	Dosage and duration	Primary and secondary outcomes	Results	Evidence
Hoffmann et al., 2003 [37]	Open-label, prospective, uncontrolled, unblinded, short follow up	36 (SP, PP)	TCA 40 mg/6 times within 3 weeks; follow up with 40 mg every 6–12 weeks; 13.1 ± 6.22, 3–23 (mean ± SD., range) months	EDSS WD	initial phase: EDSS (initial 5.6 ± 0.93 (mean ± S.D.); end: 4.9 ± 1.0; *P* < .001). WD: (initial: 294 ± 314 m; end: 604 ± 540 m; *P* < .001) follow up: EDSS and WD remained stable	IV

Hellwig et al., 2004 [38]	Open-label, prospective, uncontrolled, unblinded, short follow up	161 (RR, SP, PP)	TCA 40 mg/6 times within 3 weeks	EDSS WD SSEP	EDSS: (initial: 6.44 ± 1.06; end: 5.47 ± 1.24): WD: (initial 158.03 ± 501.20, end: 439.38 ± 895.24). SSEP latencies: reduced for all variables (*P* < .0001)	IV

Hoffmann et al., 2006 [39]	Open-label, prospective, uncontrolled, unblinded, short follow up	27 (SP, PP)	TCA 40 mg/6 times within 3 weeks	EDSS WD WT 25-*f*-test CSF changes	EDSS: (initial: 5.4 ± 1.3; end: 4.9 ± 1.1; *P* < .001). WD and WT increased: *P* < .001, 25 *f*-test increased: *P* < .01 CSF changes n.s.	IV

Hellwig et al., 2006 [40]	open-label over a 52-week long interval, prospective, randomized, unblinded	TCA: 34 (SP, PP) MIX: 30 (SP, PP)	TCA: 40 mg every 6–12 weeks, 52 weeks MIX: initial dose: 12 mg/m^2^ 2nd dose: 8–10 mg/m^2^ 6 weeks later/then quarterly: 52 weeks	EDSS WD	TCA: EDSS decreased (*P* < .001) WD: increased (*P* < .001) MIX: EDSS, WD n.s.	III

TCA: triamcinolone-acetonide acid; RR: relapsing-remitting MS; PP: primary chronic progressive MS; SP: secondarychronic progressive MS; MIX: mitoxantrone; EDSS: expanded disability status scale; WD: maximum walking distance; WT: maximum walking time; SSEP: somatosensory evoked potentials; CSF: cerebrospinal fluid; n.s: non significant.
